# Medical Convention

**Published:** 1851-09

**Authors:** 


### MEDICAL CONVENTION.

We take pleasure in publishing the following call for a convention of the Physicians of Kentucky. We trust that the call will be heartily responded to from all parts of the State, and that the meeting in Frankfort will be full, cordial, spirited, and harmonious:

*To the Medical Profession in Kentucky:*

After much deliberation and consultation, the undersigned are fully convinced that the general interests of our time-honored profession and the public welfare alike demand some concentrated association and action on our part. We therefore venture to request our brethren of every county in Kentucky to meet us in convention at Frankrort on the 1st day of October next, for the purpose of organizing a State Medical Society—to frame a constitution for the government of the same, and to take steps to procure from the Legislature such chartered rights and privileges as may be deemed necessary to give permanency and efficiency to the association.

Similar societies exist in almost every State of this confederacy—the broad and swelling streams of general and professional literature are flowing in upon us on every side—and shall we ingloriously stay or obstruct their onward course, or adopt the more noble part of contributing, if but some small rivulets, to the swelling tide? This age of progress and practical improvement will not tolerate inaction, or permit us to remain stationary—we must advance or retrograde. If we are not content to lag dishonorably far in the rear of our sister States, we must arouse ourselves to vigorous effort, and seek to concentrate and garner up, for present use and future reference, the rich fruits of daily observation, now available only to the immediate observer. The head, the heart, and the hand must co-operate in this great work—then may we hope to awaken a spirit of virtuous emulation in the profession; to protect the public from gross imposition by imparting useful information to the people at large; to draw deep and permanent the line of demarcation between the man of science and the pretender; to enlarge the sphere of our usefulness; and to build up a reputation worthy of our self-sacrificing and noble pursuit.

Permanent among the benefits that may be anticipated from an association, such as we contemplate, we may enumerate the following:

It will be the means of nurturing in the members of the profession a kindlier spirit toward each other—thrown periodically together, they will grasp the warm hand of friendship—interchange the results of thought and experience—rub off all asperities—drown petty grievances—stifle animosities, and unite in the christian and philanthropic labor of promoting harmony and good will among the members of the profession.

It will confer upon the profession all the vigor and power of concentrated and associated action.

It will be the means of diffusing more widely correct principles.

It will furnish occasion to compare the results of varying modes of treatment, and thus promote successful practice.

Arousing a spirit of inquiry and emulation to the very borders of the State, the practice of recording observations, as they occur, will become more general, and thus be the means of perpetuating much important practical information that would otherwise be lost.

It will be the most formidable obstacle to the desolating spread of knavish quackery. As some guarantee of suitable education must be required before medicine can confer upon society all the manifold blessings it is capable of dispensing, a proper effort on the part of the whole body of respectable practitioners of Kentucky may, doubtless, secure from intelligent legislators some enactment establishing a standard of qualification for those who propose to assume the responsible duty of taking charge of the public health.

It will be an efficient instrument for the correction of popular errors.

It may secure and put in permanent form the vital statistics of every county in the State.

It will give an impetus to the much-neglected study of public hygiene, and may draw the attention of the Legislature to that important subject—pointing out and directing the reforms that may be required to promote the public health.

It may make sufficient developments to satisfy our legislators of the importance—moral, political, and medical—of a systematic registration of marriages, births, and deaths.

It will accumulate accurate information in regard to the modifying influences of local causes on disease—will bring to light such diseases as may be peculiar to certain localities, and furnish the most abundunt material for a complete history of past and future epidemics.

It will secure a permanent and authoritative record of such improvements and discoveries as have been or may hereafter be made by distinguished members of the profession in this State—thus redeeming for Kentucky the honor conferred upon her by such benefactors as McDowell and Brashear, and securing to her the credit of any future achievement of her cherished sons.

It will digest a uniform code of Medical Ethics, the observance of which will, in no long time, wipe out the standing reproach that "doctors will disagree."

Such are some of the objects we propose to effect by the organization of a State Medical Society. All regular practitioners in the State are equal in rights, and are alike interested in the results that may flow from his organization, and of course ought to be consulted. We therefore invite the attention of our brethren to this important subject, and we cannot but indulge the hope that it will appear to them in the same imposing light that it does to us, and that we shall have the pleasure of meeting a representation from every county in the State, at the place and time designated.

H. S. Chipley, M.D., W. T. Shortridge, M.D., S. M. Letcher, M. D., J. S. Halstead, M.D., H. M. S. Killman, M.D., Benj. P. Drake, M.D., Jno. C. Darby, M.D., Wm. B. Cromwell, M.D., Jno. R. Desha, M.D., D, J. Ayres, M.D., Jos. G. Roberts, M.D., W. L. Sutton, M.D., Wm. H. Barlow, M.D., J. M. Barclay, M.D., P. Rankins, M. D., H. Craig, M.D., L. S. Erricson, M.D., W. B. Paxton, M.D., Jas. M. Williams, M.D., John T. Parker, M.D., Ben. Hensley, M.D., W. C. Sneed, M.D., J. M. Mills, M.D., D. W. Roper, M.D., Ben. Monroe, M.D,, E. .H Watson, M.D., Charles G. Phythian, M.D., J. B. Newman, M.D., John Mills, M.D., S. F. Gano, M.D., J. D. Winston, M.D,, John Sutton, M.D., Lloyd Warfield, M.D., J.W. Knight, M.D., U. E. Ewing, M.D., T. S. Bell, M.D.

